# Effects of chemical cues and prior experience on predator avoidance in crayfish

**DOI:** 10.1002/ece3.10426

**Published:** 2023-08-11

**Authors:** Davinder Kaur, Azeem Iqbal, Ismael Soto, Jan Kubec, Miloš Buřič

**Affiliations:** ^1^ South Bohemian Research Center of Aquaculture and Biodiversity of Hydrocenoses, Faculty of Fisheries and Protection of Waters University of South Bohemia in České Budějovice Vodňany Czech Republic

**Keywords:** alarm cues, crayfish, fish, infochemicals, kairomones, predation

## Abstract

Multisensory stimuli provide organisms with information to assess the threat present in the surroundings. Olfactory cues show dominance over other sensory modalities in the aquatic environment. The impact of chemical predator cues combined with experiences gained (learning) in species without previous contact is not fully understood. We investigated the foraging and shelter‐seeking behaviour of naïve and experienced marbled crayfish *Procambarus virginalis* juveniles in response to the chemical signals of pumpkinseed *Lepomis gibbosus* alone and in combination with alarm chemicals produced by preyed‐upon conspecifics. Naïve and experienced (previously exposed to pumpkinseed predation) juveniles were stocked in an arena with shelter and feed and exposed (1) to water from a tank containing a predator actively feeding on conspecifics, (2) water from a tank with predator only and (3) water only as control. Crayfish exposed to the combined stimuli avoided the inlet zone and gravitated to shelter zone of the arena to a greater extent than did those exposed to predator‐only cues and the control. Regardless of the treatment, experienced crayfish showed significantly reduced interest in feeding. Our findings imply that crayfish response to threat‐associated odours with the greatest potency when visual or tactile cues are present, while previous encounters with predators may make them more cautious.

## Graphical abstract

Multisensory stimuli provide organisms with information to assess the threat present in the surroundings. We investigated the foraging and shelter‐seeking behaviour of naïve and experienced marbled crayfish *Procambarus virginalis* juveniles in response to the chemical signals of pumpkinseed *Lepomis gibbosus* alone and in combination with alarm chemicals produced by preyed‐upon conspecifics. Our findings imply that crayfish response to threat‐associated odours with the greatest potency when visual or tactile cues are present, while previous encounters with predators may make them more cautious.

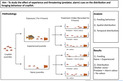

## 1 INTRODUCTION

Organisms employ a variety of means to gather pertinent information about their surroundings at both temporal and spatial scales (Moore et al., 2021). For instance, at the smallest scales, chemical signals can induce single‐celled organisms to gather, forming a super organism during reproduction (Bobek et al., 2017). Conversely, at the largest scales, Arctic terns and salmon employ the distribution of spatial and temporal cues to perform extensive orientation, facilitating their ability to locate food sources or return to their natal streams (Nevitt et al., 2008; Ueda, 2014). However, sensory stimuli reflecting abiotic and biotic characteristics can be unevenly distributed in the environment (Clark & Moore, 2018; Lunt & Smee, 2015). Animals are exposed to multisensory stimuli of varied intensity and duration throughout life (Wagner & Moore, 2022), affecting physiology (Iqbal et al., 2023), behaviour, morphology and life history (Lima & Dill, 1990; Wei & Zhang, 2022). Identifying relative adaptive value of sensory stimuli/reception is a challenge because of interactions among influencing factors.

The behaviour concept *landscape of fear* has gained attention of ecologists in the past two decades. This concept highlights the role of spatially heterogeneous predation risk in driving prey behaviour and trophic cascades through the integration of behavioural, population, community and spatial ecology disciplines. This hypothesis primarily focuses on organism behaviour when sensing predation risk (Gaynor et al., 2019). Indeed, the risk allocation hypothesis, suggest that animals can discern whether their surroundings are high or low risk with some degree of accuracy (Lima & Bednekoff, 1999). Prey can integrate visual, chemical, acoustic and tactile input (Bouwma & Hazlett, 2001; Lukas et al., 2021) to assess risk posed by the surrounding environment and alter behaviour to avoid danger. The hermit crab *Pagurus bernhardus*, for example, shows its highest level of anti‐predator behaviour when exposed to chemical and visual cues simultaneously (Dalesman & Inchley, 2008). The sulphur molly *Poecilia sulphuraria* reacts to a unique combination of acoustic and visual stimuli produced by aquatic predators (Lukas et al., 2021). An organism's likelihood of devoting time and energy to potentially metabolically expensive avoidance measures depends on its ability to perceive and identify threats.

In the aquatic environment, turbidity, habitat complexity, water flow and light conditions can limit visual perception, making chemical communication the most reliable mode of accessing information about the risk of predation (Dalesman & Inchley, 2008; Wisenden, 2000). Kairomones (predatory cues) can enter the water from predator skin, gills and/or excrement (Brown et al., 1995; Glover et al., 2013), alerting prey to predator location, identity and motivational state (Häberli et al., 2005). The association of kairomones with alarm cues emitted by prey under attack, can enhance the fright response among conspecifics (Ramberg‐Pihl & Yurewicz, 2020). The checkered periwinkle *Littorina scutulata* exhibits risk hierarchical behaviour, escalating from predator response to alarm response to a combination of the two (Keppel & Scrosati, 2004). As most predators are generalists and opportunistic feeders, conspecific alarm scent can provide more precise information of the prevailing risk.

Experience and the learning process impact success of predator avoidance. Familiar conspecific alarm scent combined with predator cues usually elicits a robust anti‐predator response including reduced activity and/or feeding as described in northern crayfish *Faxonius virilis* and damselfish *Pomacentrus amboinensis* (Acquistapace et al., 2003; Holmes & McCormick, 2010). Prey can make decisions based on the available sensory signals influenced by prior experience.

Crayfish, primarily exhibit nocturnal behaviour, heavily rely on their well‐developed chemoreception system for communication (Kubec et al., 2019; Little, 1975; Schmidt & Mellon, 2010; Zulandt Schneider & Moore, 2000). Research has shown that crayfish utilise chemical stimuli to deduce information about their diet (Beattie & Moore, 2018), gape ratio, gape size (Wood & Moore, 2020) and the level of threat posed by potential predators (Wagner & Moore, 2022). As recognised keystone species in ecosystems, crayfish play a crucial role in shaping the environment (Creed & Reed, 2004). Alterations in their behaviour can have direct and indirect consequences on the ecosystems they inhabit, emphasising their ecological significance. Therefore, the obligate parthenogenetic invasive marbled crayfish *Procambarus virginalis* Lyko 2017 is an ideal single species model organism (Hossain et al., 2018; Vogt, 2011) due to the elimination of genotype‐related variability (Martin et al., 2007; Vogt et al., 2008).

In Europe, the pumpkinseed sunfish *Lepomis gibbosus* Linnaeus, 1758, has successfully established itself as one of the most prevalent non‐native freshwater fish species spreading across various countries from north to south (Fox et al., 2007; Sterud & Jørgensen, 2006). It has a strong invasive potential and adaptable life‐history, as shown by the predictive model of pumpkinseed invasiveness (Copp & Fox, 2007). It also serves as a potential daytime predator of juvenile crayfish, leading to a notable impact on the prey population (Tetzlaff et al., 2011).

As both species are invasive in European waters (Lipták & Vitázková, 2014; Ondračkovć et al., 2011), it's crucial to study how the pumpkinseed sunfish and marbled crayfish interact as predators and prey. Investigating their relationship, especially involving scent and prior learning, is essential. Despite existing studies are more focused on crayfish behaviour related to the cues (Kenison et al., 2018; Pecor et al., 2010; Ramberg‐Pihl & Yurewicz, 2020), the combined effect of cues and previous experiences in their interactions are overlooked. Understanding this complexity can provide valuable insights into the interactions of non‐native species in the ecosystem.

The objective of this study was to characterise the response of a prey organism to chemical stimuli involving predator cues and conspecific alarm scent in naïve versus experienced prey. We hypothesise that the experienced prey would reduce feeding in the presence of predator cues only, while the naïve group with no prior contact with the predator would react to a combination of predator and conspecific alarm scent.

## 2 MATERIALS AND METHODS

### 2.1 Animals

Marbled crayfish originated from the culture at the Research Institute of Fish Culture and Hydrobiology in Vodňany, FFPW USB, Czech Republic. Two thousand seven hundred juveniles at developmental stage 4/5 were randomly selected from culture tanks. Weight to nearest 0.1 mg was obtained using an analytical balance (Kern & Sohn GmbH). Each individual was used only once during the experiment. The mean weight of specimens (7.4 ± 0.04 mg) did not differ among treatments.

Sixty pumpkinseed were collected from fishponds near to Vodňany (FFPW) at University of South Bohemia (USB), Czech Republic and held in outdoor tanks at ambient temperature and photoperiod until acclimation to experimental conditions. Fish were fed with chironomid larvae and had no prior experience with crayfish. Total length (cm), standard length (cm) and weight (g) were measured using a ruler and electronic balance (Kern & Sohn GmbH). Each fish was used only once during the experiment. There was no significant difference in size and weight among treatment groups (Table 1).

**TABLE 1:** Total length (TL), standard length (SL) and weight (W) of pumpkinseed *Lepomis gibbosus* (*n* = 60) used in trials with predator cues plus alarm scent (P + A) and only predator cues (P).

Fish (predator)	P + A	P	*t*‐Test	*p*‐Value
TL (cm)	8.1 ± 0.7	8.2 ± 0.7	0.716	0.477
SL (cm)	6.6 ± 0.6	6.7 ± 0.6	10.575	0.295
W (g)	9.0 ± 4.0	9.6 ± 3.4	0.753	0.454

*Note*: There were no significant differences among groups (*t*‐test). Data are presented as mean ± standard deviation.

Both species are listed in the European Commission Regulation (EU Regulation No. 1143/2014 and Commission Implementing Regulation No. 2016/1141) that prohibits their manipulation, holding and breeding except for their eradication and research purposes. Extreme caution was exercised to prevent escape of animals into the natural environment.

Experimental procedures including rearing, capture and measurements were conducted according to the principles of the Ethical Committee for the Protection of Animals in Research of the USB, FFPW, Research Institute of Fish Culture and Hydrobiology, Vodňany, based on the EU harmonised animal welfare act of Czech Republic. The Institutional Animal Care and Use Committee (IACUC) approved this study and the principles of laboratory animal care and national laws 246/1992 and regulations on animal welfare were followed (Ref. No. 22761/2009‐17210).

### 2.2 Experimental design

All animals were acclimatised at 20°C in aerated tanks (460 × 320 × 220 mm) for 24 h before experimentation. Marbled crayfish juveniles used in the experiment were categorised as naive or experienced based on their previous exposure to the predator. The group of crayfish categorised as ‘experienced’ was created by exposing crayfish to the fish predator for 4 h, beginning 24 h prior to the start of the experiment. During the exposure period, both crayfish and fish could move freely about the tank (with airstone). Predator was actively feeding on the juveniles during this period, therefore shelter (bricks) was equipped in the tank to ensure a sufficient number of surviving crayfish. A naïve group had no previous contact with the predator.

**FIGURE 1:**
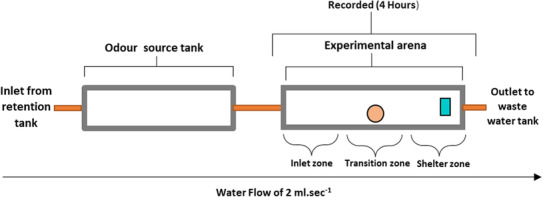
Experimental system. The odour source tank (on the left side) was the source of specific infochemicals directed with water flow through silicon tubes into the experimental arena. The experimental arena contained 20 juvenile marbled crayfish, feed (orange circle) and shelter (blue rectangle). For the analysis of crayfish movement, segments of the experimental arena were considered inlet, transition and shelter zone. Water flowed from the retention tank through odour source tank and experimental arena to the wastewater tank. The water flow was maintained at 2 mL s^−1^, resulting in the complete system's water being removed twice during the 4‐h recording period.

The experimental system consisted of two tanks supplied with dechlorinated tap water from a retention tank at 20°C connected by silicon tubes and pumps with flow regulation. Water was pumped from the odour source tank (400 × 200 × 250 mm, 15 L aged tap water), through a plastic pipe that supplied the experimental arena (190 × 140 × 75 mm, 2 L aged tap water), and subsequently to a wastewater tank. The outlets of the odour source tank and experimental arena were covered with plastic mesh to prevent crayfish escape (Figure 1). The experimental arena contained a halved ceramic plant pot (18 mm depth, 53 mm entry) as shelter at the downstream end. Frozen chironomid larvae as feed were placed in the centre of the experimental arena. The water flow was maintained at 2 mL s^−1^, resulting in complete water renewal twice during the 4‐h recording period. This duration allowed enough time for transferring the information from the predator to the prey.

The experimental design consisted of three treatments differing in the stock of the infochemical odour source tank: (1) a single pumpkinseed with 30 marbled crayfish to provide predator cues and crayfish alarm scent (P + A) from an actively foraging predator and prey, (2) single pumpkinseed without prey to obtain predator cues only (P) and (3) tap water only as control (C). The predatory fish were starved for 48 h before the trials to ensure effective predation and stocked 10 min prior to experimentation. We exposed naïve and experienced juveniles marbled crayfish to each above mentioned treatment in experimental arenas. Randomly selected 20 juveniles were stocked in the experimental arena 5 min prior to start of experiment. At the beginning of the trial, 40 chironomid larvae were placed in the centre of the experimental arena, and activity was video recorded for 4 h. Each treatment was replicated 15 times with naïve and experienced juveniles {Naïve individuals (no. of trails = 45) exposed to treatment P + A (no of trails = 15), P (no of trails = 15) and C (no of trails = 15); Experienced individuals (no. of trails = 45) exposed to treatment P + A (no of trails = 15), P (no of trails = 15) and C (no of trails = 15)}. Trials were conducted in light from fluorescent tubes (2310 lm) from 10.00 to 14.00 h to avoid diurnal effects. Use of six experimental systems enabled observation of response to treatment of naïve and experienced crayfish simultaneously.

### 2.3 Feed consumption analysis

Chironomid larvae were thawed at room temperature, drained through a sieve lined with filter paper to remove excess water and weighed prior to placement in the experimental arenas. At the end of the trial, remaining larvae were removed using a plastic pipette, sieved as previously and weighed using an electronic analytical balance (Kern & Sohn GmbH) to the nearest 0.1 mg. The consumption of feed per individual mass (%) was calculated as follow:

$$\text{Chironomous consumption}\;\mathrm{per}\;\text{individual mass}=\frac{\text{Initial chironomid mass}-\text{Final chironomid mass}}{\text{Juvenile crayfish mass}}\times 100.$$

### 2.4 Crayfish behaviour analysis

Activity was recorded for 4 h using digital video cameras (HDR‐CX240E, Sony) attached above the experimental arenas. A still photo was taken from the recordings every 5 min, for a total of 48 images for the observations. The arena was subjectively visualised as inlet, transition and shelter zones for the purposes of movement analysis (Figure 1). The juveniles were counted in each zone for the spatial (refers to the overall number of juveniles in each zone during the 4‐h trail period) and temporal (refer to the change in the number of juveniles in each zone with time) distribution.

### 2.5 Statistical analysis

To assess differences in larva consumption rate among groups [Treatment (P + A/P/C) and Exposure (Experienced/Naïve)], we performed the non‐parametric Kruskal–Wallis test because the assumptions for parametric tests were not satisfied.

To analyse the distribution of crayfish in each zone and preference for a given zone over time, we performed generalised linear mixed model (GLMM) using the glmer function of the lme4 R package (Bates et al., 2015). Nine models were created to understand the effect of predictor (exposure, treatment and time) on the response variables: three models of spatial distribution (one per zone), and six models of temporal distribution (one per zone per treatment and exposure) using the number of crayfish as response variable in each zone. We selected the negative binomial family as suitable for count data after inspecting the distribution of the data and residuals through histograms. *Arena* was specified as random effect to account for the dependence of the within group observations in each arena, thus producing more accurate estimates of the fixed effects (i.e. number of crayfish).

## 3 RESULTS

### 3.1 Consumption rate

**FIGURE 2:**
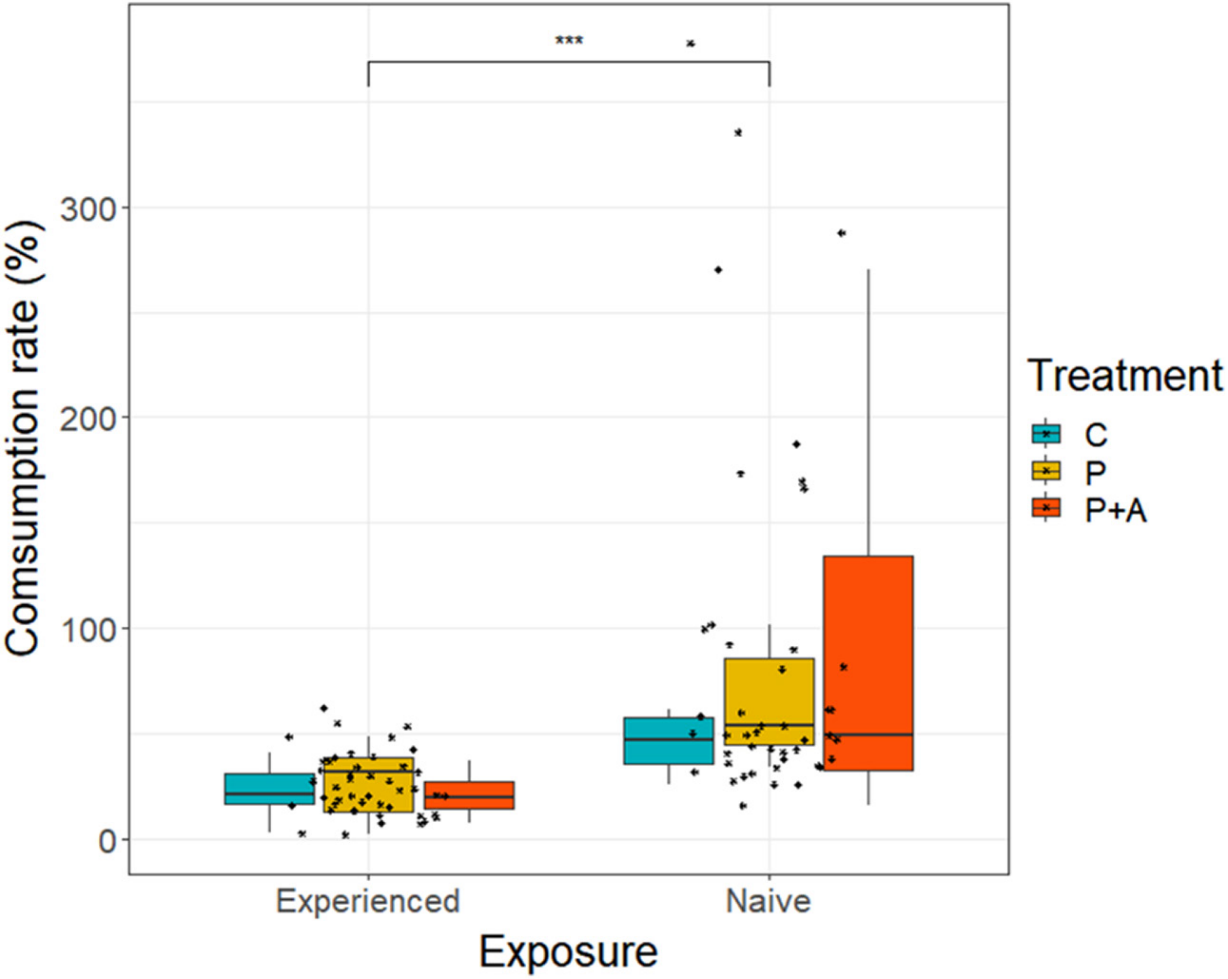
Rate of chironomid larvae consumption (%) of juvenile marbled crayfish with different level of exposure (Experienced and Naïve) and treatment groups (P + A = predator cues + alarm scent treatment, P = predator cues only, C = control). Asterisk (***) represents the significance level between naive and experienced group (*p* < .001).

Our result shows that feeding was significantly affected by prior exposure/learning. The experienced juveniles (*χ* ^2^ = 36.192, *p* < .05, Figure 2) consumed significantly less feed than the naïve group. No effect of treatment on consumption rate was observed (*χ* ^2^ = 2.047, *p* > .05, Figure 2). No significant interaction among exposure (Experienced, Naïve) and treatment (P + A, P, C) was observed.

### 3.2 Spatial distribution—Overall

**FIGURE 3:**
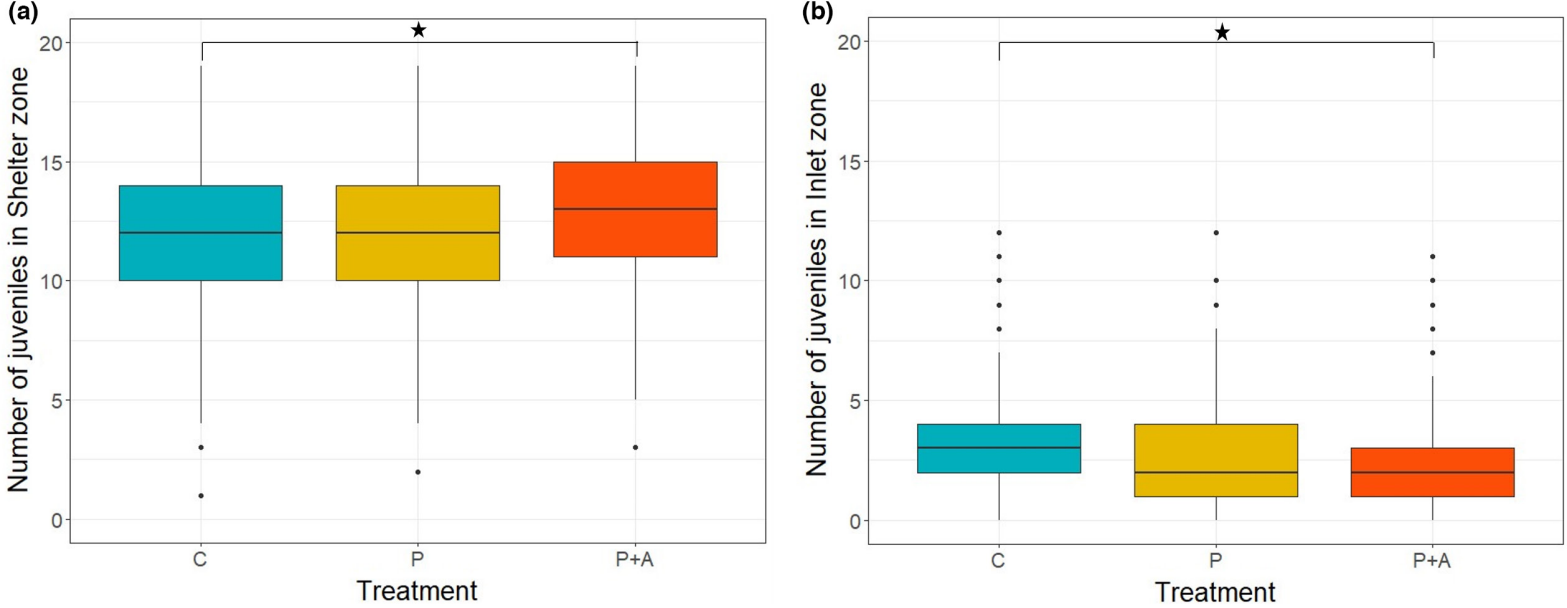
Number of marbled crayfish juveniles observed in the shelter zone (a) and inlet zone (b) among treatment groups (P + A = predator cues + alarm scent treatment, P = predator cues only, C = control). Asterisk (*) represents the significance level between treatment P + A and C (*p* < .05).

Differences in crayfish spatial distribution were found for P + A, with significantly more crayfish observed in the shelter zone (*Z*_treatment_ = 2.071, *p* < .05, Table S2) and fewer occupying the inlet zone (*Z*_treatment_ = −1.930, *p* = .05, Figure 3). Predator cues alone and prior exposure showed no significant impact on crayfish distribution. A non‐significant trend of higher numbers of naïve crayfish in the transition zone was observed (*Z*_exposure_ = 1.656, *p* > .05, Figure S2).

### 3.3 Temporal patterns of spatial distribution

**FIGURE 4:**
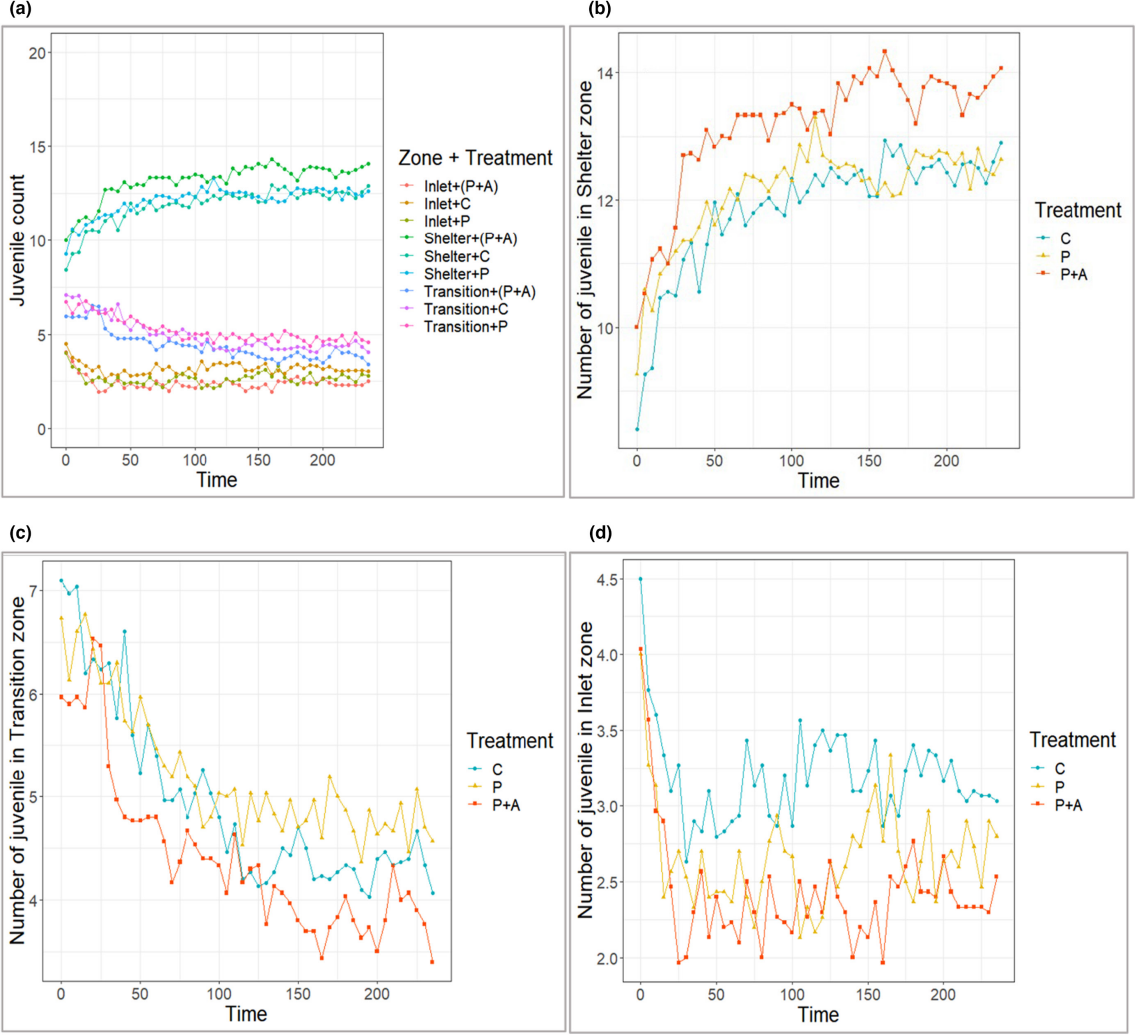
Mean temporal distribution of marbled crayfish juveniles relative to treatment (P + A = predator cues + alarm scent, P = predator cues only, C = control): (a) overall distribution among all zones and in particular zones—(b) shelter zone, (c) transition zone and (d) inlet zone of the experimental arena.

**FIGURE 5:**
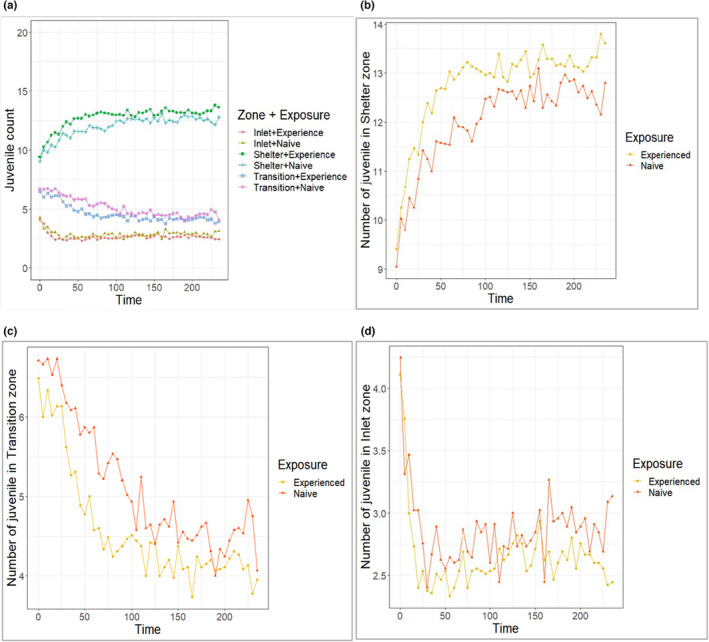
Mean temporal distribution of naïve and experienced marbled crayfish juveniles; (a) overall distribution in all zones and in particular zones—(b) shelter zone, (c) transition zone and (d) inlet zone of the experimental arena.

Neither treatment nor prior exposure/learning showed a significant effect on the temporal pattern of spatial distribution of crayfish in the arena, although we observed a trend of higher numbers of crayfish of P + A treatment occupying the shelter zone compared to transition and inlet zones (Figure 4). The similar trend was seen in experienced crayfish compared to naïve (Figure 5). Overall, crayfish distribution was dramatically skewed to the shelter zone (*Z*_time(treatment)_ = 3.629, *p* < .05, *Z*_time(exposure)_ = 2.401, *p* < .05) in the initial 50 min, becoming stabilised or rising more slowly for the remainder of the recorded period. A contrasting trend was observed with a decline in transition zone occupancy (*Z*_time(treatment)_ = −3.885, *p* < .05, *Z*_time(exposure)_ = −3.678, *p* < .05). No overall temporal effect on distribution was found for the inlet zone, although a sharp decline was noted in the initial 30 min (Figures 4 and 5).

## 4 DISCUSSION

Organism survival depends on its ability to recognise and alter its behaviour in response to predation threat (Brown & Laland, 2003; Sih, 1986). Although anti‐predatory responses can differ at individual, community and species level, they usually involve decline in foraging, feed consumption and/or increased shelter use (Holmes & McCormick, 2010; Wagner & Moore, 2022). The aim of this study was to investigate how a prey organism responds to chemical stimuli, specifically predator cues and conspecific alarm scent, and compare the behaviour of naïve and experienced prey. We observed marbled crayfish juveniles to significantly increase shelter use in the presence of a predator combined with conspecific alarm cues. A more pronounced pattern was seen in the reduced feeding by juvenile crayfish that had been previously experienced, no matter if under conditions of combined effect, predator cues only or even if there was no cue, which were in contradiction with our hypothesis that the experienced prey would decrease their feeding behaviour when exposed to predator cues alone. Our findings suggest that foraging rate and spatio/temporal distribution is affected by previous experience with predation to a greater extent than by infochemicals. Only experience with a predator was related to increased caution, while naïve juveniles changed behaviour only slightly in accordance to infochemicals with no visual or tactile stimuli.

### 4.1 Impact of cues

Previous studies have shown decreased foraging (Gherardi et al., 2011) and increased shelter seeking (Beattie & Moore, 2018; Gherardi et al., 2011; MacKay et al., 2021) in crayfish exposed to cues associated with the predator and/or active predation of conspecifics. Our results showed crayfish juveniles to forage less and be more likely to remain in the shelter zone in the presence of both predator and alarm cues than when exposed to predator cues only and control treatment. Similarly, when *Orconectes virilis* were exposed to both an unlearned alarm cue and a predator cue simultaneously, they develop a learned association perceiving it as elevated risk of predation (Hazlett et al., 2002). The ignorance of the predator cue alone could indicate low risk unless it is associated with the predation of conspecifics (Beattie & Moore, 2018; Lima & Bednekoff, 1999). However, permanent predator odour can be associated with increase in consumption rate in prey, possibly an adaptation to accelerate growth to exceed the predator gape limits (Wood & Moore, 2020). Research has shown that the presence of predators can stress potential prey, leading to the voluntary release of disturbance cues (Bairos‐Novak et al., 2019).

The presence of cues did not have an impact on the feed consumption of juveniles. Nevertheless, when it came to naïve animals exposed to predator and alarm cues or predator cues alone, there was a notable inconsistency in their feeding responses. This variability may be attributed to individual differences in personality (Galib et al., 2022) or potentially the existence of an unidentified cue.

In addition to foraging/feeding behaviour, the spatial distribution of crayfish in the experimental arena was monitored. During the 4‐h experimental period, crayfish moved throughout the arena with increasing density in the shelter zone over time in all treatments. The initial 50 min showed maximum shift, followed by a gradual increase. Lima and Bednekoff (1999), in a risk allocation hypothesis, stated that prey behaviour is driven by temporal variation in predation risk. Prey can possess the accurate perception of their risk level, by utilising the cues to determine their current level of threat. In our study, the risk cues were constant in the predator‐only treatment but increased in the P + A treatment with ongoing predation in the odour source tank. Therefore, juveniles choose to stay in the shelter to avoid predation, thus increasing the probability of their survival. However, the crayfish response was unexpectedly low in the predator only treatment, possibly because of the lack of visual or tactile cues to reinforce the threat perception. The absence of visual contact or predator‐generated turbulence detected by mechanoreceptors could minimise the perceived threat, leading to the shift in preference and again approaching feed after several minutes. Overall, time had no significant impact on the distribution in the inlet zone, but an acute reduction in crayfish presence was observed in the initial 30 min.

The P + A group juveniles spent the least time in the inlet zone, significantly differing from control. Our results agree with those of previous studies, confirming that, in a threatening sensory landscape, prey will avoid risky areas (landscape of fear) and move towards safer places (landscape of safety; Gaynor et al., 2019; MacKay et al., 2021). The control juveniles showed a similar pattern, even in the lack of threat, perhaps initially attracted by feed and, when satiated, the shelter was the primary attractant as a basic crayfish resource to protect them from predation and cannibalism (Holdich, 2002; Kubec et al., 2019).

The current study focused on the threat perceived by predator plus alarm cues compared with predator cues only, as well a control scenario with no chemical cues. Generally, a combination of all these cues are necessary to signal the existing risk to conspecifics (Smith, 1992), but the most important factor affecting the response of juvenile crayfish in the present study was the level of the predator knowledge risk.

### 4.2 Impact of prior exposure/learning

Studies have identified the impact of learning/experience on prey defence strategies in fish and snails (Bairos‐Novak et al., 2019; Larson & McCormick, 2005; Turner et al., 2006). Results of the current study suggest that learning is a factor when an organism experiences direct encounters with a predator (visual + chemical + mechanical cues). This encounter allows the prey to sense and recall danger more efficiently when the threatening odour is in the general vicinity. Prior exposure to a predator is particularly significant in juveniles, as this period is characterised by high predation and mortality (Gosselin & Qian, 1997; Momot, 1984). The speed with which information is acquired and acted upon can greatly affect the population density and distribution in space (Brown, 2003; Wisessnden et al., 1997). Our study showed the reactions of experienced crayfish to be more pronounced, including those exposed to predator cues only and the controls. Experienced crayfish showed significantly reduced feeding compared to the naïve group regardless of treatment but exhibited no difference in shelter use. The effect of learning lead to prey experience that may have induced anxiety or stress that led to decreased feeding and vigilance in a novel environment for an extended period. The duration of the potential effect is not known, but it may well be anchored in memory for the reported recall duration of crayfish (Kubec et al., 2019; Tierney & Andrews, 2013).

Numerous studies have looked at how different environments (ponds and rivers) influence crayfish behaviour (Bergman et al., 2006; Hossain et al., 2020). This suggests that learning associations may vary between pond and river ecosystems. In ponds, the same sequence strengthens the formation of a learned association, whereas, in streams, when predator odour and alarm odour are presented together, learning associations are blocked (Hazlett, 2003). But this blocking learning irrelevance case would be for fast flowing environment, in which the removal of the cues is at faster pace. In our study, a minimal flow (2 mL s^−1^) was enough for inducing a significant impact on the prey.

## 5 CONCLUSION

Within social groups in the aquatic environment, chemical cues play an important role in acquiring and transferring information among individuals (Ferrari et al., 2007a, 2007b). In this study, the efficacy with which the prey acted on the innate and acquired knowledge of the chemically indicated potential threat suggests that chemical communication plays an essential role in predator/prey interaction, but the type and strength of reaction can be affected by experience and the other sensory inputs, influencing behaviour. The duration of the impact of increased attentiveness or anxiety on crayfish behaviour and its interaction with visual and tactile stimuli is worth investigating. Such factors play a role in the establishment of invading species such as marbled crayfish that have been shown to exhibit high adaptability and represent a threat to ecosystems.

## AUTHOR CONTRIBUTIONS

**Davinder Kaur:** Conceptualization (equal); formal analysis (equal); investigation (equal); methodology (equal); visualization (equal); writing – original draft (equal). **Azeem Iqbal:** Investigation (equal); methodology (equal); writing – review and editing (equal). **Ismael Soto:** Data curation (equal); formal analysis (equal); writing – review and editing (equal). **Jan Kubec:** Conceptualization (equal); investigation (equal); methodology (equal); writing – review and editing (equal). **Miloš Buřič:** Conceptualization (equal); formal analysis (equal); funding acquisition (equal); methodology (equal); supervision (equal); visualization (equal); writing – review and editing (equal).

## CONFLICT OF INTEREST STATEMENT

The authors declare that they have no competing interest.

## Supporting information


Appendix S1

Appendix S2

Appendix S3

## Data Availability

Data are publicly available through the Dryad repository: https://doi.org/10.5061/dryad.w3r2280wk.
