# Supplementary material for: Effects of chemical cues and prior experience on predator avoidance in crayfish
Source: Ecol Evol. 2023 Aug 11;13(8):e10426. doi: 10.1002/ece3.10426 (PMC10421732; doi:10.1002/ece3.10426)
Supplement: Supplementary file 3 — Appendix S3 [file ECE3-13-e10426-s001.docx]

**Spatial and Temporal Distribution**

| **Fixed Effects** | Estimate | Std. Error | z value | Pr(>\|z\|) |
| --- | --- | --- | --- | --- |
| (Intercept) | -0.51891 | 0.04586 | -11.316 | <2e-16 *** |
| Time1 | 0.18504 | 0.07708 | 2.401 | 0.0164 * |
| ExposureNaïve | 0.08775 | 0.06483 | -1.354 | 0.1759 |
| Time1:ExposureNaïve | 0.03873 | 0.10800 | 0.359 | 0.7199 |

**Table S1**: Results of glmer model in context to the number of juveniles in response to time and exposure in the shelter zone.

**Table S2** : Results of glmer model in context to the number of juveniles in response to time and treatment in the shelter zone.

| **Fixed effects** | Estimate | Std. Error | z value | Pr(>\|z\|) |
| --- | --- | --- | --- | --- |
| (Intercept) | -0.62872 | 0.04543 | -13.839 | < 2e-16 *** |
| Time2 | 0.24186 | 0.06664 | 3.629 | 0.000284 *** |
| TreatmentP | 0.06485 | 0.07166 | 0.905 | 0.365523 |
| TreatmentP+A | 0.13166 | 0.06357 | 2.071 | 0.038336 * |
| Time2:TreatmentP | -0.07817 | 0.11337 | -0.690 | 0.490505 |
| Time2:TreatmentP+A | -0.03545 | 0.09567 | -0.371 | 0.710976 |

**Table S3** : Results of glmer model in context to the number of juveniles in response to exposure and treatment in the shelter zone.

| **Fixed effects** | Estimate | Std. Error | z value | Pr(>\|z\|) |
| --- | --- | --- | --- | --- |
| (Intercept) | 2.47340 | 0.05057 | 48.910 | <2e-16 *** |
| ExposureNaïve | -0.03149 | 0.07142 | -0.441 | 0.6593 |
| TreatmentP | 0.03928 | 0.05979 | 0.657 | 0.5112 |
| TreatmentP+A | 0.13390 | 0.07829 | 1.710 | 0.0872 . |
| ExposureNaïve:TreatmentP | -0.02780 | 0.08251 | -0.337 | 0.7362 |
| ExposureNaïve:TreatmentP+A | -0.05364 | 0.11399 | -0.471 | 0.6379 |

**Table S4** : Results of glmer model in context to the number of juveniles in response to time and exposure in the transition zone.

| **Fixed effects** | Estimate | Std. Error | z value | Pr(>\|z\|) |
| --- | --- | --- | --- | --- |
| (Intercept) | -1.00503 | 0.07048 | -14.260 | < 2e-16 *** |
| Timet1 | -0.42874 | 0.11657 | -3.678 | 0.000235 *** |
| ExposureNaïve | 0.13864 | 0.09747 | 1.422 | 0.154915 |
| Timet1:ExposureNaïve | -0.03625 | 0.16236 | -0.223 | 0.823330 |

**Table S5** : Results of glmer model in context to the number of juveniles in response to time and treatment in the transition zone.

| **Fixed effects** | Estimate | Std. Error | z value | Pr(>\|z\|) |
| --- | --- | --- | --- | --- |
| (Intercept) | -0.888814 | 0.079092 | -11.238 | < 2e-16 *** |
| Timet2 | -0.505473 | 0.130125 | -3.885 | 0.000103 *** |
| TreatmentP | -0.020737 | 0.117673 | -0.176 | 0.860120 |
| TreatmentP+A | -0.111936 | 0.111755 | -1.002 | 0.316529 |
| Timet2:TreatmentP | 0.159971 | 0.196633 | 0.814 | 0.415903 |
| Timet2:TreatmentP+A | 0.000816 | 0.183605 | 0.004 | 0.996454 |

**Table S6** : Results of glmer model in context to the number of juveniles in response to exposure and treatment in the transition zone.

| **Fixed effects** | Estimate | Std. Error | z value | Pr(>\|z\|) |
| --- | --- | --- | --- | --- |
| (Intercept) | 1.45681 | 0.06688 | 21.781 | <2e-16 *** |
| ExposureNaïve | 0.15110 | 0.09127 | 1.656 | 0.0978 . |
| TreatmentP | 0.08880 | 0.09580 | 0.927 | 0.3540 |
| TreatmentP+A | -0.13580 | 0.10036 | -1.353 | 0.1760 |
| ExposureNaïve:TreatmentP | -0.03398 | 0.13071 | -0.260 | 0.7949 |
| ExposureNaïve:TreatmentP+A | 0.07946 | 0.13614 | 0.584 | 0.5594 |

**Table S7** : Results of glmer model in context to the number of juveniles in response to time and exposure in the inlet zone.

| **Fixed effects** | Estimate | Std. Error | z value | Pr(>\|z\|) |
| --- | --- | --- | --- | --- |
| (Intercept) | -1.52775 | 0.10454 | -14.615 | <2e-16 *** |
| Timei1 | -0.08259 | 0.15592 | -0.530 | 0.596 |
| ExposureNaïve | 0.05199 | 0.14728 | 0.353 | 0.724 |
| Timei1:ExposureNaïve | 0.05449 | 0.22106 | 0.246 | 0.805 |

**Table S8** : Results of glmer model in context to the number of juveniles in response to time and treatment in the inlet zone.

| **Fixed effects** | Estimate | Std. Error | z value | Pr(>\|z\|) |
| --- | --- | --- | --- | --- |
| (Intercept) | -1.3549 | 0.1095 | -12.37 | <2e-16 *** |
| Timei2 | -0.0421 | 0.1600 | -0.26 | 0.79 |
| TreatmentP | -0.1986 | 0.1588 | -1.25 | 0.21 |
| TreatmentP+A | -0.2389 | 0.1670 | -1.43 | 0.15 |
| Timei2:TreatmentP | 0.0329 | 0.2348 | 0.14 | 0.89 |
| Timei2:TreatmentP+A | -0.0778 | 0.2528 | -0.31 | 0.76 |

**Table S9** : Results of glmer model in context to the number of juveniles in response to exposure and treatment in the inlet zone.

| **Fixed effects** | Estimate | Std. Error | z value | Pr(>\|z\|) |
| --- | --- | --- | --- | --- |
| (Intercept) | 1.04587 | 0.10229 | 10.224 | <2e-16 *** |
| ExposureNaïve | 0.03936 | 0.14742 | 0.267 | 0.7895 |
| TreatmentP | -0.23382 | 0.14355 | -1.629 | 0.1033 |
| TreatmentP+A | -0.31563 | 0.16353 | -1.930 | 0.0536 * |
| ExposureNaïve:TreatmentP | 0.08177 | 0.20646 | 0.396 | 0.6921 |
| ExposureNaïve:TreatmentP+A | 0.02523 | 0.23350 | 0.108 | 0.9140 |

**Chironomus (food) consumption per individual (%)**

**Table S10** : Results of Kruskal Wallis test in context to the consumption of Chironomus larva in the presence of treatment and exposure.

| **Type** | Kruskal-Wallis chi-squared | df | p-value |
| --- | --- | --- | --- |
| Treatment | 2.047 | 2 | 0.3593 |
| Exposure | 36.192 | 1 | 1.788e-09*** |

**Table S11** : Rate of chironomid larvae consumption (%) of experienced and naïve juvenile marbled crayfish in presence of predator cues (P), predator cues + alarm scent (P+A), and in control (C). Data are presented as mean ± standard deviation.

| **Exposure groups** | **Treatment groups** | | |
| --- | --- | --- | --- |
|  | P+A | P | C |
| Naïve | 85.7 ± 74.6 ^a^ | 85.1 ± 74.5 ^a^ | 84.3 ± 99.9 ^a^ |
| Experienced | 22.5 ± 11.7 ^c^ | 28.3 ± 15.0 ^b^ | 26.7 ± 15.4 ^bc^ |

**SUPPLEMENTARY FIGURE 1** : Distribution of juveniles in transition zone


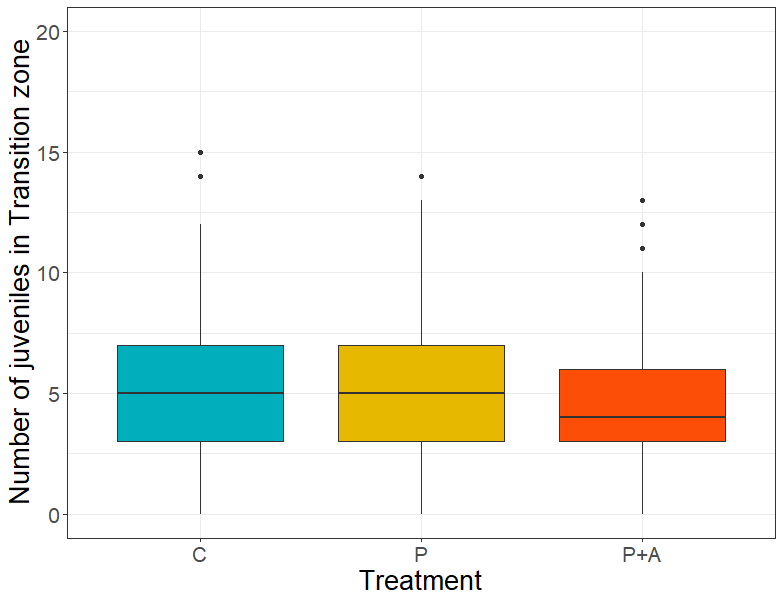


**Fig. S1** : The comparison of the frequency in marbled crayfish juveniles in the transition zone among treatment groups (P+A = predator cues + alarm odour treatment, P = predator cues only, C = control).

**SUPPLEMENTARY FIGURE 2** : Distribution of naive and experienced juveniles in different zones


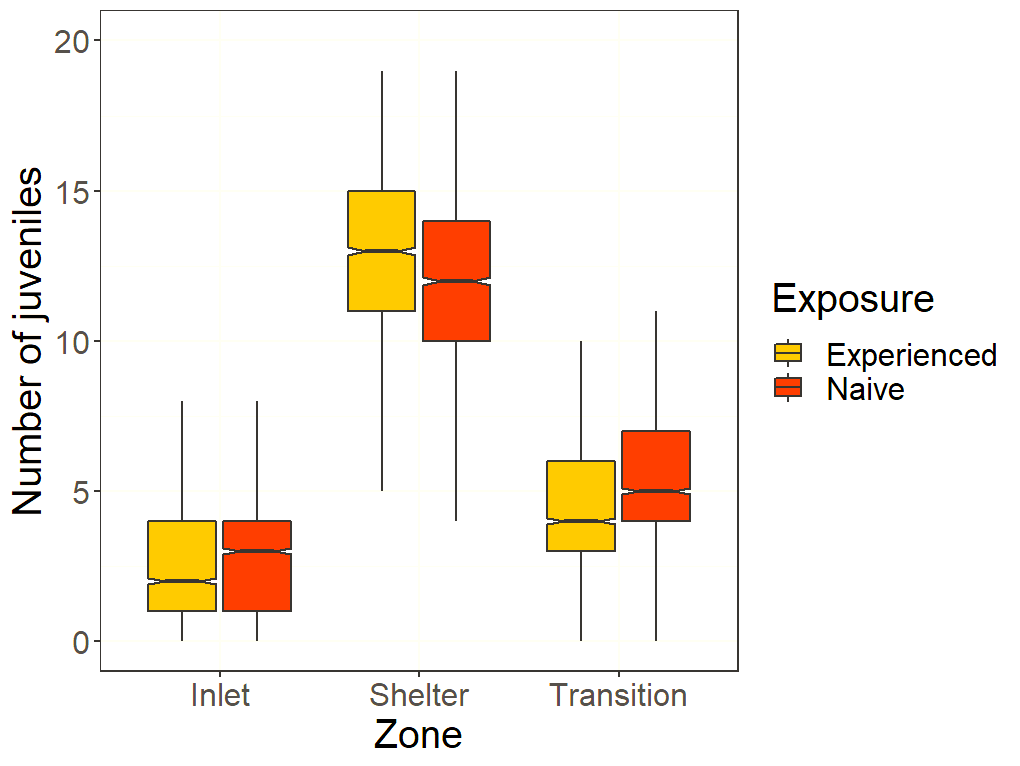


**Fig. S2** : The comparison of the frequency in naive and experienced marbled crayfish juveniles in the different zones.
